# A randomised, blinded, controlled field study to assess the efficacy and safety of lotilaner tablets (Credelio™) in controlling fleas in client-owned dogs in European countries

**DOI:** 10.1186/s13071-017-2479-8

**Published:** 2017-11-01

**Authors:** Daniela Cavalleri, Martin Murphy, Wolfgang Seewald, Jason Drake, Steve Nanchen

**Affiliations:** 1 Elanco Animal Health, Mattenstrasse 24a, 4058 Basel, Switzerland; 2 Elanco Animal Health, 2500 Innovation Way, Greenfield, IN 46140 USA

**Keywords:** Lotilaner, Credelio, Fleas, Fipronil, Frontline, Field, Dog, Efficacy, Safety, Europe

## Abstract

**Background:**

Lotilaner is a novel isoxazoline developed for oral administration to dogs. In laboratory studies, lotilaner was shown to be safe and to produce a rapid flea and tick knockdown, with a sustained speed of kill for at least a month post-treatment. A study was undertaken to demonstrate the efficacy, safety and palatability of three monthly doses of lotilaner flavoured chewable tablets (Credelio™, Elanco) in controlling fleas under field conditions in Europe.

**Methods:**

Dogs were enrolled at 17 veterinary clinics across Germany, Hungary and Portugal. Qualifying households having no more than three dogs and one primary dog with at least five fleas was randomised 2:1 to a lotilaner (minimum dose rate 20 mg/kg) or a topical fipronil group (administered per label). There were 128 and 64 households allocated to the lotilaner and fipronil groups, respectively. Treatments were dispensed to owners on Days 0, 28 and 56; supplementary household dogs received the same treatment as the primary dog. Post-enrollment flea counts and flea allergy dermatitis (FAD) assessments were made on primary dogs on Days 14, 28, 56 and 84. Efficacy calculations were based on geometric mean percent reductions of live flea counts *versus* pre-treatment counts on Day 0. The safety and palatability of lotilaner tablets were also assessed.

**Results:**

Lotilaner efficacy was 99.1, 99.5, 99.9 and 99.8% on Days 14, 28, 56 and 84, respectively. Corresponding reductions for fipronil were 93.4, 91.2, 94.4 and 97.0%. Lotilaner was superior to fipronil at all post-Day 0 assessments (*t*_(186)_ ≥ 3.43, *P* ≤ 0.0007). At every post-treatment assessment, at least 90% of lotilaner-treated dogs were flea-free (98.4% on Day 84); fewer than 90% of fipronil group dogs were flea-free on the same time points. Lotilaner flavoured chewable tablets were palatable, and both products were well tolerated. Lotilaner alleviated or eliminated clinical signs of FAD, including pruritus.

**Conclusions:**

Under field conditions in Europe, lotilaner flavoured chewable tablets were greater than 99% effective in eliminating fleas from dogs at the first post-treatment assessment (Day 14). Efficacy was maintained through Day 84, with corresponding improvements in FAD. Lotilaner tablets were palatable and safe and provided superior flea control to fipronil.

**Electronic supplementary material:**

The online version of this article (10.1186/s13071-017-2479-8) contains supplementary material, which is available to authorized users.

## Background

The European release of spinosad in 2011 as a monthly orally administered flea adulticide for dogs heralded a trend away from topical products to oral formulations to control fleas. That trend was recently accelerated by the release of a novel family of compounds, the isoxazolines, that target distinct binding sites on γ-aminobutyric acid- and glutamate-gated chloride channels [1]. Laboratory and field studies of the three isoxazolines - afoxolaner, fluralaner and sarolaner - that were granted initial approvals for oral administration to dogs in 2014 and 2015, demonstrated that these compounds were safe and effective, and provided 1 month (afoxolaner and sarolaner) to two to 3 months (fluralaner) activity against fleas and ticks [2–4].

Lotilaner is a novel isoxazoline that was selected for development from a library of over 500 structures emerging from a research program to identify compounds that would be effective against ectoparasites of pets. Laboratory testing in dogs demonstrated that the minimum lotilaner dose rate of 20 mg/kg rapidly began to kill fleas and the tick *Ixodes ricinus*, with efficacy demonstrated from two and 4 h after treatment, respectively [5, 6]. This early onset of action aligned with expectations from pharmacokinetic work which showed that lotilaner achieves peak blood levels in dogs within approximately 2 h after treatment, and has a half-life of approximately 30 days, thereby sustaining flea and tick activity for at least 1 month after treatment [7–9]. A study involving repeated monthly administrations of lotilaner to puppies from 8 weeks of age at doses of up to 5 times the upper limit of the product dose range demonstrated that lotilaner has a wide safety margin [10]. These promising laboratory data needed translation into real-world clinical benefits, and the study reported herein was initiated to assess the performance of lotilaner in client-owned dogs naturally infested with fleas.

The main objective of this study was to evaluate the safety and efficacy of lotilaner flavoured chewable tablets (Credelio™) against flea infestations on dogs in Europe. Lotilaner was administered orally by owners once every 4 weeks for a total of three treatments at the minimum dose rate of 20 mg/kg body weight to dogs naturally infested with fleas in three countries, Germany, Hungary and Portugal. A topical formulation of fipronil (Frontline® Spot-on) was used as a positive control comparator. The effect of treatment on clinical signs (pruritus, erythema, scaling, papules, alopecia, and pyoderma) associated with flea allergy dermatitis (FAD) and the palatability of the oral product was also evaluated; dogs were also observed for any adverse events.

## Methods

This assessor-blinded, positive controlled, randomised, multicentre, non-inferiority clinical field study was conducted in compliance with the principles of Good Clinical Practices and with the guidelines of the World Association for the Advancement of Veterinary Parasitology [11, 12].

### Animals

To qualify for inclusion, a household could have no more than three dogs and two cats provided that dogs and cats did not contact each other or did not share resting places for the duration of the study. At least one household dog was required to have an infestation of at least five fleas, and all household dogs were required to be at least 8 weeks of age and weigh at least 2 kg. The first household dog presenting with an infestation of at least five fleas was the primary dog on which all study assessments would be used for effectiveness calculations. All household dogs were required to be healthy or to have conditions judged by the investigating veterinarian as not likely to interfere with the objectives of the study. The owner or authorised agent was required to provide informed consent as a condition of enrolment.

A household was excluded from the study if it contained dogs that were convalescent, or if there were dogs that were pregnant or lactating, or that were intended for breeding until 4 months following the last treatment administration. Households would be removed from the study at any time at the discretion of the investigator or study sponsor for reasons that included protocol non-compliance (for instance, treatment with a study-proscribed product such as one that had any effectiveness against fleas), the appearance of concomitant disease, or development of a serious adverse event that was incompatible with continuation in the study.

Non-primary dogs in each household were treated with the same product as the primary dogs, and data from these supplementary dogs were included for assessments of palatability (for lotilaner) and safety. Cats and other non-study animals in enrolled households were treated during the study with a commercial ectoparasiticide, efficacious against fleas, provided by the enrolling clinic. These animals were not involved in any efficacy, palatability or safety evaluations.

All dogs were kept with their owners under their usual housing conditions before, during and after the study. Because one of the study products was applied topically, bathing/immersion in water within 2 days after application and more frequent bathing than once a week was to be avoided. Dogs were not allowed to swim in watercourses for 2 days after application, and any water contact was to be documented by the owner.

### Randomisation and treatment

Within each clinic, dogs were randomised per household to the treatment groups in the sequence of inclusion designated by the randomization plan, using a block design and a 2:1 ratio (lotilaner:fipronil), with a targeted total enrolment of 180 households. The first household dog presenting with an infestation of at least five fleas was the primary dog on which all study assessments would be used for effectiveness calculations. All dogs, including supplementary household dogs, were observed for any adverse events.

All dogs from any household were randomised to the same treatment group: Group 1 households were dispensed lotilaner flavoured chewable tablets (Credelio™, Elanco, Basel, Switzerland), available in five tablet sizes (56.25 mg, 112.5 mg, 225 mg, 450 mg and 900 mg), to be administered on the basis of each household dog’s body weight to achieve a minimum dose of 20 mg/kg. At the initial visit and at the second and third visits, the dispenser in each clinic provided the appropriate number of tablets for each household dog to be treated on a single occasion on each of Days 0, 28 (± 2) and 56 (± 2). Owners were instructed to feed their dogs within 30 min prior to treatment. Group 2 households were dispensed a formulation of fipronil 10% (Frontline® Spot on, Merial), available in three sizes (0.67 ml, 1.34 ml or 2.68 ml), for at-home application on each of Days 0, 28 (± 2), and 56 (± 2).

### Study assessments

Whole-body flea counts were completed on primary dogs on Days 0, 14 (± 2), 28 (± 2), 56 (± 2) and 84 (± 2). Each primary dog was combed thoroughly, according to a specific sequence, for at least 10 min, continuing until at least 5 min after the last flea was found. Flea counts were censored at 101; i.e. all counts above 100 were recorded as “more than 100”. In the statistical analysis, these values were treated as 101. There were very few such values (all but one occurring at baseline; one occurred in the fipronil group at the Day 28 assessment, so any bias caused by treating these values as 101 should be in favour of fipronil). Fleas that were retrieved were placed into sealable plastic bags, stored deep-frozen at approximately minus 18 °C and forwarded to a laboratory for speciation using established morphological keys [13, 14].

Physical examinations, body weight measurements and FAD assessments were completed on each primary dog at each visit. Blood and urine samples were collected for clinical pathology evaluations on Days 0 and 84 (or earlier for dogs prematurely exiting the study).

The three study populations for assessments were: the safety population, consisting of all dogs, primary and supplementary, that were randomised to a treatment group and that received at least one dose of either study product; the intent-to-treat (ITT) population consisting of all primary dogs in each treatment group; and the per-protocol (PP) population, consisting of all primary dogs without major protocol violations. The analyses of efficacy were based on the ITT population, and assessments were also conducted to compare with the results of the PP population.

The effectiveness of each treatment was assessed by comparing baseline flea comb counts on Day 0 with those scheduled at 14 (± 2), 28 (± 2), 56 (± 2), and 84 (± 2) days after the first treatment administration. Efficacy was determined on the basis of the percent reduction in flea counts from pre- to post-dosing within each treatment group. Percent effectiveness at each counting timepoint after dosing was calculated as follows:

$$ \mathrm{Percent}\  \mathrm{efficacy}=\left(\left(\mathrm{MB}-\mathrm{MA}\right)/\mathrm{MB}\right)\times 100 $$

where MB is the mean flea count prior to dosing (Day 0) and MA is the mean flea count post-dosing (Day 28, 56 and 84).

Calculations were performed using geometric and arithmetic means. Calculation of geometric means involved taking the logarithm of the flea count of each dog. If any of the flea counts were equal to zero, a one was added to the count for every animal in the group and then subtracted from the resultant mean prior to calculating percent effectiveness.

For flea counts, treatment groups were compared by analysis of (co)variance (AN(C)OVA) methods if the assumption of normal distribution was satisfied on the original scale or after possible log transformation. In the ANCOVA, the number of dogs per household was used as a covariate. Non-inferiority was claimed if the two-sided 95% confidence interval (CI) for the ratio of flea counts (or FAD scores) for lotilaner, divided by the same value for fipronil, lay completely within the interval (0, 1/0.85) or (0, 1.17), providing 97.5% confidence that flea counts (or FAD scores) from lotilaner treatment were not higher than flea counts (or FAD scores) from fipronil treatment, up to a non-inferiority margin of 15%. Superiority was claimed if the 95% CI lay completely within the interval (0, 1), providing 97.5% confidence that flea counts (or FAD scores) from lotilaner treatment were lower than flea counts (or FAD scores) from fipronil treatment. FAD scores were also compared to baseline with the Wilcoxon test for paired samples.

Each primary dog was graded for six clinical signs of FAD - pruritus, erythema, scaling, papules, alopecia, and dermatitis/pyodermatitis - using a 4-point scale: 0 (absent); 1 (mild); 2 (moderate); and 3 (severe) [15, 16]. For pruritus, the scoring was: 0, none/no scratching; 1, occasional scratching; 2, frequent scratching and/or self-biting; and 3, intense scratching/biting. The FAD score calculated for each primary dog at each time point was the sum of the clinical sign scores and when data were adequate, total FAD scores were to be analysed within treatment group over time and/or at each time point, and between the treatment groups.

For sex, age, body weight, breed, hair length, husbandry, animal spends time indoor/outdoor, summary statistics and/or frequencies were calculated. The two groups were compared with a non-parametric test (Kruskal-Wallis for breed and hair length, Mann-Whitney for age and body weight, and Fisher’s exact test for sex, husbandry, animal spends time indoor/outdoor).

The acceptance rate of lotilaner flavoured tablets was defined as the number of all successful dosings, divided by the number of all dosings, times 100. Palatability was determined according to the guideline of the European Medicines Agency, Committee for Medicinal Products for Veterinary Use, based on acceptance when the lotilaner flavoured tablet was offered to the dog in an empty bowl or trough, or on the ground during 60 s, or if the tablet was accepted when offered by hand for an additional 60 s [17].

### Translation

French translation of the Abstract is available in Additional file 1. 

## Results

### Animals and treatments

One hundred and ninety-two primary dogs (households) were enrolled into the study, 128 in the lotilaner group and 64 in the fipronil group, at five clinics in Germany, five clinics in Hungary, and seven clinics in Portugal. Clinics were geographically dispersed across each country. Including supplementary dogs in each household, the safety population comprised 180 dogs treated with lotilaner and 91 with fipronil. Owners reported administering treatments per schedule, and all treatments were successfully administered by owners. Across the safety population, the administered lotilaner dose rate ranged from 20.1 to 40.7 mg/kg. Eighty percent of lotilaner treatments were voluntarily accepted either from an empty food bowl or on the ground, or from the hand. There were no owner reports of study dogs being exposed to water within 2 days after application, being bathed or swimming in water courses, or having water contact during the study.

Groups were homogeneous for age, weight and sex distribution, and there were no statistically significant baseline differences between treatment groups in sex, age, body weight, breed, hair length, husbandry, and whether the primary dog spent most time indoors/outdoors (Table 1). Most dogs in each group came from single-dog households (Table 2). Forty-one different breeds were included in the study, of which the most frequently enrolled were Labrador retriever (*n* = 7), Yorkshire terrier (*n* = 7), American Staffordshire terrier (*n* = 5), Beagle (*n* = 4), Boxer (*n* = 4) and Spaniel (non-specific) (*n* = 4).

**Table 1:** Demographics of enrolled dogs (efficacy population)

		Lotilaner (*n* = 128)	Fipronil (*n* = 64)
Age (years)	Mean ± SD	4.6 ± 3.3	4.9 (3.9)
	Range	0.2–15.0	0.2–15.0
Weight (kg)	Mean ± SD	16.7 ± 11.4	17.5 (13.3)
	Range	2.4–54.2	2.2–62.1
Sex	Female	60 (46.9%)	31 (48.4%)
	Male	68 (53.1%)	33 (51.6%)
Primary dog spends time	Mostly indoors	52 (40.6%)	29 (45.3%)
	Mostly outdoors	76 (59.4%)	35 (54.7%)

*Abbreviation*: *SD* standard deviation

**Table 2:** Number of households with cats and numbers of dogs in households

	Lotilaner *n* (%)	Fipronil *n* (%)
Number of households	128	64
With 0 cats	93 (72.7)	44 (68.8)
With 1 cat	11 (8.6)	7 (10.9)
With 2 cats	24 (18.8)	13 (20.3)
With 1 dog	93 (72.7)	43 (67.2)
With 2 dogs	18 (14.1)	15 (23.4)
With 3 dogs	17 (13.3)	6 (9.3)
Total number of dogs	180	91

One primary dog in the lotilaner group was removed from the study on Day 68 following its death due to being hit by a car. A supplementary dog in this group was also removed (on Day 41) following its death due to a traffic accident. A supplementary dog in the fipronil group died suddenly on Day 81, with a tentative diagnosis of cardiac failure due to infarction. Data from the primary dog that died were included in all calculations except for Day 84. Because the ITT and PP numbers and the statistical values for all comparisons were almost identical, only the ITT results are reported herein.

### Flea efficacy assessments

**Fig. 1:**
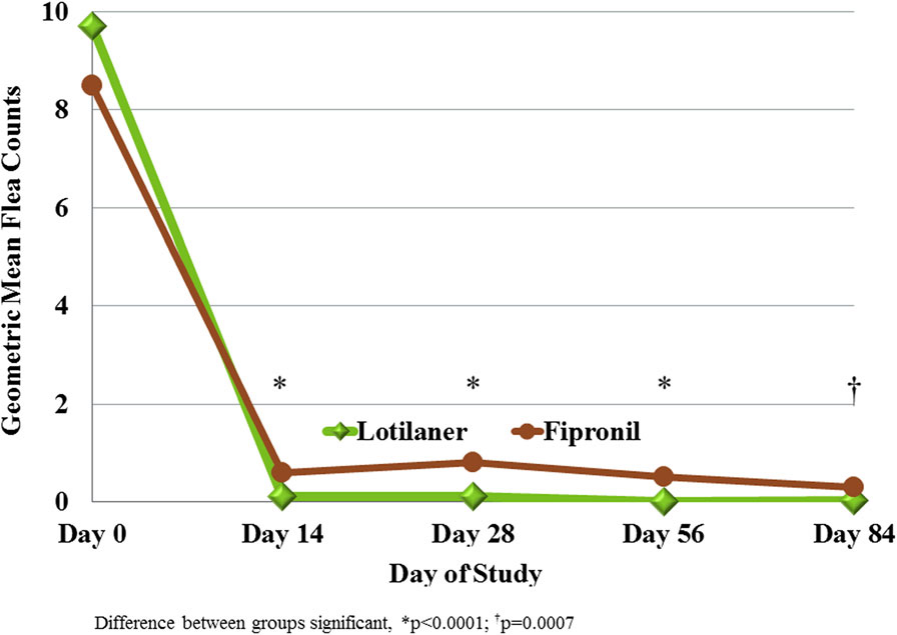
Geometric mean flea counts of lotilaner and fipronil-treated dogs at each study assessment. Difference between groups significant: *P < 0.0001; †*P* = 0.007

Baseline geometric mean flea counts in the lotilaner and fipronil groups were 9.7 and 8.5, respectively (Table 3; Fig. 1). Of the fleas retrieved at baseline from dogs in the lotilaner group that could be speciated, 77.8% were *C. felis* and 19.9% were *Ctenocephalides canis*. The equivalent numbers in the fipronil group were 77.3% and 19.1%. Low numbers of *Pulex irritans* and *Archaeopsylla erinacei* were collected from dogs randomised to each group, and three *Nosopsyllus fasciatus* fleas were identified from dogs randomised to the lotilaner group. None of these fleas was present at the final evaluation. Reports from participating clinics described the regular presentation of flea-infested dogs from non-study households, verifying that conditions during the study period were conducive to a flea challenge.

**Table 3:** Flea count data for each treatment group

		Lotilaner	Fipronil	95% Confidence interval Lotilaner/Fipronil
Day 0	Arithmetic mean ± SD	15.0 ± 22.0	11.7 ± 16.2	Not applicable
	Range^a^	5–101	5–101	
	Geometric mean	9.7	8.5	
Day 14	Arithmetic mean ± SD	0.2 ± 0.6	2.7 ± 11.1	0.57–0.80
	Range	0–4	0–86	
	Geometric mean	0.1	0.6	
Day 28	Arithmetic mean ± SD	0.1 ± 0.7	4.9 ± 15.7	0.47–0.71
	Range^a^	0–8	0–101	
	Geometric mean	0.1	0.8	
Day 56	Arithmetic mean ± SD	0.01 ± 0.1	2.3 ± 8.4	0.57–0.78
	Range	0–1	0–58	
	Geometric mean	0.01	0.5	
Day 84	Arithmetic mean ± SD	0.04 ± 0.3	1.4 ± 6.9	0.70–0.91
	Range	0–3	0–53	
	Geometric mean	0.02	0.3	

*Abbreviation*: *SD* standard deviation

^a^Any count > 100 was assigned the value 101

Geometric mean flea counts of the lotilaner group were compared to those of the fipronil group for non-inferiority, with a 15% margin, for each time point during the study. Non-inferiority was demonstrated because the two-sided 95% confidence interval for the ratio of mean flea counts for fleas for the lotilaner group, divided by the same value for the fipronil group, lay completely within the interval (0, 1/0.85) or (0, 1.17). The statistical analyses also demonstrated the superiority of lotilaner in reducing geometric mean flea counts compared to fipronil at all post-Day 0 assessments (Tables 3 and 4). For the lotilaner group, across all post-Day 0 assessments, the overall percent reductions in mean flea counts (*C. canis* and *C. felis*) in the lotilaner group were 99.5% (arithmetic mean) and 99.6% (geometric mean) (Table 4). For the fipronil group, the equivalent reductions were 75.9 and 94.1%, respectively.

**Table 4:** Percent flea count reduction from baseline for each treatment group

	Effectiveness based on	Lotilaner	Fipronil	*t*-value	*P*-value
Day 14	Arithmetic mean	99.0	77.3		
	Geometric mean	99.1	93.4	*t*_(187)_ = 4.61	*P* < 0.0001
Day 28	Arithmetic mean	99.3	58.0		
	Geometric mean	99.5	91.2	*t*_(187)_ = 5.33	*P* < 0.0001
Day 56	Arithmetic mean	99.9	79.9		
	Geometric mean	99.9	94.4	*t*_(187)_ = 5.01	*P* < 0.0001
Day 84	Arithmetic mean	99.7	88.4		
	Geometric mean	99.8	97.0	*t*_(186)_ = 3.43	*P* = 0.0007
Overall	Arithmetic mean	99.5	75.9		
	Geometric mean	99.6	94.1	*t*_(187)_ = 5.69	*P* < 0.0001

Both treatments had brought about reductions in flea counts within the 14 days following the first treatment, with 100% of lotilaner-treated dogs and 85.9% of fipronil-treated dogs with fewer than five fleas, and 90.6 and 76.6% of dogs in each group free of fleas, respectively (Table 5). Of the six dogs with baseline (Day 0) flea counts of at least 100 fleas, five were randomised to the lotilaner group, and all were free of fleas at the end of the study. The one fipronil-group dog with a Day 0 count of at least 100 fleas remained infested at each assessment, with one flea found on Day 84. On Day 84, in the lotilaner group, all but two dogs were free of fleas: the two dogs had burdens of two fleas and three fleas. On this occasion, fleas were found in nine fipronil-group dogs, six dogs had fewer than five fleas, and in three dogs counts were 10, 15 and 53 fleas. The baseline counts in these dogs were 19, 13 and 14, respectively.

**Table 5:** Percent of dogs in each group with zero fleas and with less than five fleas. Missing values not included in assessment

		Day of study			
		14	28	56	84
Dogs with 0 fleas	Lotilaner	90.6	95.3	99.2	97.7
	Fipronil	76.6	75.0	81.3	85.9
Dogs with < 5 fleas	Lotilaner	100	99.2	100	100
	Fipronil	85.9	85.9	87.5	95.3

**Fig. 2:**
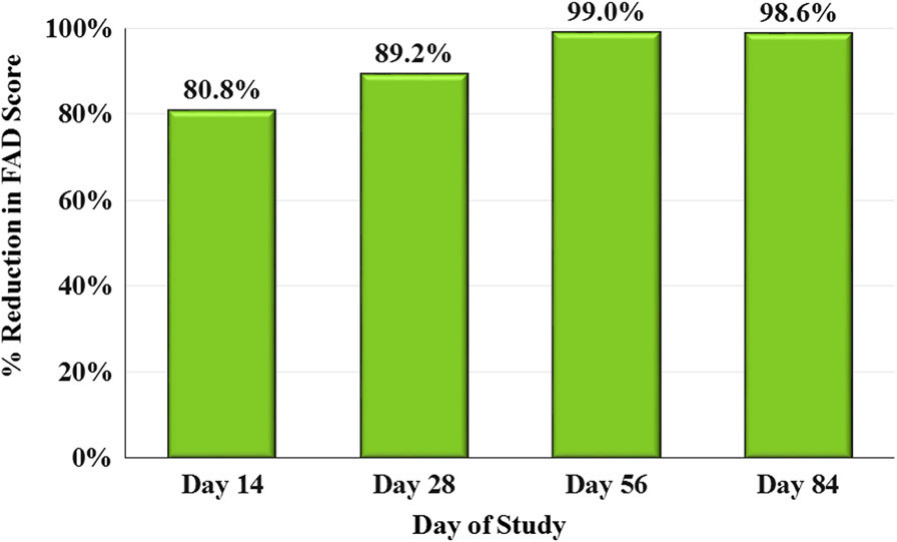
Percent reduction in lotilaner group dogs of mean total score of flea allergy dermatitis (fipronil group was not assessed because too few dogs were affected at baseline)

**Fig. 3:**
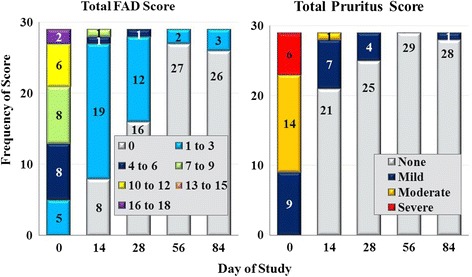
Frequency of total flea allergy dermatitis (FAD) and pruritus scores in the 29 lotilaner-group dogs that were affected at baseline

Baseline (Day 0) FAD was diagnosed in 29 (22.7%) lotilaner-group dogs, but in only four (6.3%) dogs randomised to the fipronil group. There were no significant between-group baseline differences for any clinical sign of FAD (i.e. pruritus, erythema, scaling, papules, alopecia and pyoderma). No further FAD analyses were applied to the fipronil group because of the low baseline incidence. For the lotilaner group, there was a significant decrease from baseline in all scores at all time points after Day 0 (Wilcoxon signed-rank test: *Z* = 9.53, *P* ≤ 0.0001). On Day 0 the mean total FAD score was 7.3, which declined to 0.8 by Day 28, after which no dogs in the group had a total score greater than 1, and an overall percent reduction in total mean FAD score of 98.6% on Day 84 (Fig. 2). At baseline, only five of the 29 dogs diagnosed with FAD had total FAD scores less than 5; by Day 56 no dogs had a score greater than 3 (Fig. 3). Pruritus scores followed the same pattern as the FAD scores, and on Day 84 only one dog was reported with pruritus, with a score of 1 (mild) (Fig. 3).

### Safety

The only serious adverse events reported from dogs in either group were the deaths resulting from traffic accidents described earlier. All other events were transient, mild to moderate in severity, all dogs recovered, none were directly attributed to either treatment, and none led to the withdrawal of dogs from the study.

The mean body weight of each treatment group did not change notably during the study period, nor did individual clinical pathology parameters, except for transient and inconsequential excursions from the normal range of blood values in three lotilaner-treated dogs (1.7%), and one fipronil-treated dog (1.1%). Serum chemistry results showed a significant (*t*_(164)_ = 2.28, *P* = 0.0236) difference for cholesterol with higher mean values in the lotilaner group on day 84, but the mean value was within the normal reference range and was not associated with any clinical changes. Urinary analysis findings (pH and specific gravity) were unremarkable and of no clinical relevance.

Vaccinations and concomitant medications were administered to lotilaner-group dogs with no associated adverse events. Concomitant medications included benazepril, cephalexin, enrofloxacin, furosemide, levothyroxine, pimobendan, praziquantel/pyrantel/febantel, and spironolactone. At one clinic, 10 dogs presenting on Day 0 with FAD (including one with a “hot spot”) were prescribed a short course of prednisolone and amoxicillin/clavulanic acid for between three and 10 days.

## Discussion

The results of this study substantiate the effectiveness of lotilaner flavoured chewable tablets, administered at the minimum dose rate of 20 mg/kg, in eliminating flea infestations from naturally-infested dogs, and in providing ongoing effectiveness over the 4 weeks following treatment. Whether for one, two or three consecutive treatments at four-week intervals, the results demonstrate that not only was lotilaner non-inferior to fipronil, as shown by the lower 97.5% confidence interval at all post-Day 0 assessments, but in fact was significantly more effective (*t*_(186)_ ≥ 3.43, *P* = 0.0007) than fipronil at each assessment. The effectiveness of lotilaner treatment against flea burdens was evident at the first post-treatment assessment on Day 14 when over 90% of treated dogs were free of fleas, arithmetic mean flea counts were reduced by 99%, and there were substantial improvements in signs of FAD.

Improvements in FAD were observed at the first post-treatment assessment, with an overall reduction in mean total FAD scores of 80.8% by Day 14, increasing to 99.0% on Day 56 and 98.6% on Day 84. The Day 0 prednisolone treatments dispensed at one clinic to alleviate the signs of FAD in eight dogs allocated to the lotilaner group would likely have contributed to the improvement seen at Day 14. The absence of FAD relapse in these dogs and the ongoing reductions in FAD in all study dogs, with similar reductions in pruritus scores, indicate the dog health benefits of lotilaner administered as part of a treatment strategy to control FAD signs.

Lotilaner has been shown to have sustained activity against fleas for at least 5 weeks post-treatment, consistently killing 100% of newly infesting fleas within 12 h, before fleas would have begun egg-laying [5, 9, 18]. Thus by quickly killing fleas, lotilaner rapidly eliminates a source of irritation, while at the same time causing a progressive depletion in the biomass of developing flea stages in the dog’s environment.

The continued presence of fleas in nine of 64 primary dogs that received fipronil, including four dogs in which final flea counts were higher than at baseline, one of which had a flea count of 53 fleas, is consistent with a similar failure of fipronil efficacy reported from a study completed in the United States [19]. Reasons for the failure of fipronil (or any topical product) have been attributed to difficulties in extruding the entire dose from the package, ensuring that entire contents are deposited on the skin and not just superficially on the hair, and restraining the treated dog sufficiently to ensure there is no product run-off. Alternatively, the possibility of emerging resistance to fipronil cannot be ignored, given the widespread use of this compound over the last 20 years, identification of resistance in the brown dog tick *Rhipicephalus sanguineus*, and the well-established recognition of resistance in agricultural pests [20, 21]. Further investigation is therefore warranted to determine the cause of fipronil treatment failures in client-owned dogs.

The ongoing findings of fleas in fipronil-treated dogs and the clinic reports of non-study dogs presenting with flea infestations during the study period indicate that the reductions in flea burdens in the lotilaner-group dogs were due to the effectiveness of treatment in conditions that were conducive to flea challenge. The identification of both *C. felis* and *C. canis* at enrollment and the elimination of these fleas during the course of the study demonstrate that lotilaner is effective against both these flea species. The very few transient adverse events observed in lotilaner-treated dogs, none of which were directly attributed to treatment, verify the safety of lotilaner when administered by clients.

## Conclusions

The results of this study, undertaken in a diverse range of client-owned dogs, demonstrates that under a wide range of real-world conditions in Europe, lotilaner flavoured chewable tablets are palatable and well tolerated by dogs. A single treatment resulted in a 99.1% reduction from baseline in geometric mean (arithmetic mean 99.0%) flea counts, and three consecutive treatments at 28-day intervals sustained that level of reduction, significantly outperforming a topical fipronil product administered according to the same schedule. The high efficacy shown by lotilaner resulted in a substantial reduction in, or elimination of signs of flea allergy dermatitis, including reductions in pruritus.

## Additional file

Additional file 1: French translation of the Abstract. (PDF 51 kb)

## Abbreviations

AN(C)OVA: analysis of (co)variance
CI: confidence interval
FAD: flea allergy dermatitis
ITT: intent to treat
PP: per protocol
SD: standard deviation
VICH: International Cooperation on Harmonisation of Technical Requirements for Registration of Veterinary Medicinal Products
