# Supplementary material for: A randomised, blinded, controlled field study to assess the efficacy and safety of lotilaner tablets (Credelio™) in controlling fleas in client-owned dogs in European countries
Source: Parasit Vectors. 2017 Nov 1;10:526. doi: 10.1186/s13071-017-2479-8 (PMC5664837; doi:10.1186/s13071-017-2479-8)
Supplement: Additional file 1: — French translation of the Abstract. (PDF 51 kb) [file 13071_2017_2479_MOESM1_ESM.pdf]

Étude de terrain randomisée, contrôlée, menée en aveugle afin d'évaluer l'efficacité et l’innocuité des comprimés de lotilaner (Credelio™) dans le contrôle des puces chez des chiens de compagnie en Europe

Daniela Cavalleri[1], Martin Murphy[1], Wolfgang Seewald[1], Jason Drake[2]* et Steve Nanchen[1]

[1]Elanco Santé animale, Schwarzwaldallee 215, CH-4058 Bâle, WRO-1032.2.58, Suisse
[2]Elanco Santé animale, 2500 Innovation Way, Greenfield, IN 46140, États-Unis

*Correspondance : drake_jon_j@elanco.com

Adresse électronique :
Daniela Cavalleri : cavalleri_daniela_a@elanco.com ;
Martin Murphy : murphy_martin_gerard@elanco.com ;
Wolfgang Seewald : seewald_wolfgang@elanco.com ;
Jason Drake : drake_jon_j@elanco.com ;
Steve Nanchen : nanchen_steve@elanco.com

Résumé

Contexte : Le lotilaner est un nouvel agent de la classe des isoxazolines développé sous forme de comprimés administrés par voie orale chez le chien. Des études conduites en laboratoire ont montré l'innocuité du lotilaner ainsi que son effet « knockdown » rapide sur les puces et tiques, et la persistance de cette activité pendant au moins un mois après le traitement. Une étude de terrain a été menée afin de démontrer l'efficacité, l’innocuité et l'appétence de trois administrations mensuelles de comprimés à croquer aromatisés à base de lotilaner (Credelio™, Elanco) dans le contrôle des puces en conditions réelles en Europe.

Méthodes : Des chiens ont été recrutés dans 17 cliniques vétérinaires en Allemagne, en Hongrie et au Portugal. Les foyers sélectionnés, ne comptant pas plus de trois chiens dont un chien principal hébergeant au moins cinq puces, ont été randomisés selon un rapport 2/1 dans un groupe lotilaner (dose minimale de 20 mg/kg) ou un groupe fipronil topique (administré conformément à la notice). Au total, 128 et 64 foyers ont respectivement été affectés aux groupes lotilaner et fipronil. Les traitements ont été délivrés aux propriétaires les J0, J28 et J56. Les autres chiens de foyers ont reçu

le même traitement que celui du chien principal. Après l'inclusion, un comptage des puces et une évaluation de la dermatite par allergie aux piqûres de puces (DAPP) ont été réalisés chez les chiens principaux à J14, J28, J56 et J84. L'efficacité était déterminée par le calcul du pourcentage moyen (moyenne géométrique) de diminution du nombre de puces vivantes par rapport au comptage réalisé à J0 préalablement au traitement. L'innocuité et l'appétence des comprimés de lotilaner ont également été évaluées.

Résultats : L'efficacité du lotilaner a respectivement atteint 99,1 %, 99,5 %, 99,9 % et 99,8 % à J14, J28, J56 et J84. Les diminutions correspondantes observées sous fipronil étaient de 93,4 %, 91,2 %, 94,4 % et 97,0 %. Le lotilaner s'est avéré supérieur au fipronil lors de toutes les évaluations réalisées après J0 ($t_{(190)} \geq 3{,}43$, $p \leq 0{,}0007$). Lors de chaque évaluation réalisée après le traitement, au moins 90 % des chiens traités par lotilaner ne présentaient aucune puce (98,4 % à J84), alors que moins de 90 % des chiens du groupe fipronil ne présentaient aucune puce lors des mêmes évaluations. Les comprimés à croquer aromatisés à base de lotilaner étaient appétents et les deux produits ont été bien tolérés. Le lotilaner a soulagé ou éliminé les signes cliniques de DAPP, y compris le prurit.

Conclusions : Dans des conditions réelles d'infestation rencontrées en Europe, les comprimés à croquer aromatisés à base de lotilaner ont présenté une efficacité supérieure à 99 % pour éliminer les puces des chiens lors de la première évaluation réalisée après le traitement (J14). Cette efficacité a persisté jusqu'à J84 et a été associée à une amélioration des signes de DAPP. Les comprimés de lotilaner se sont avérés appétents et bien tolérés et ont permis un meilleur contrôle des puces que le fipronil.
